# Bacterial-Epithelial Contact Is a Key Determinant of Host Innate Immune Responses to Enteropathogenic and Enteroaggregative *Escherichia coli*

**DOI:** 10.1371/journal.pone.0027030

**Published:** 2011-10-28

**Authors:** Lindsey A. Edwards, Mona Bajaj-Elliott, Nigel J. Klein, Simon H. Murch, Alan D. Phillips

**Affiliations:** 1 Centre for Paediatric Gastroenterology, Royal Free Hospital, London, United Kingdom; 2 Infectious Diseases and Microbiology, Institute of Child health, London, United Kingdom; 3 Division of Metabolic and Vascular Health, Warwick Medical School, Coventry, United Kingdom; National Institute of Environmental Health Sciences, United States of America

## Abstract

**Background:**

Enteropathogenic (EPEC) and Enteroaggregative (EAEC) *E. coli* have similar, but distinct clinical symptoms and modes of pathogenesis. Nevertheless when they infect the gastrointestinal tract, it is thought that their flagellin causes IL-8 release leading to neutrophil recruitment and gastroenteritis. However, this may not be the whole story as the effect of bacterial adherence to IEC innate response(s) remains unclear. Therefore, we have characterized which bacterial motifs contribute to the innate epithelial response to EPEC and EAEC, using a range of EPEC and EAEC isogenic mutant strains.

**Methodology:**

Caco-2 and HEp-2 cell lines were exposed to prototypical EPEC strain E2348/69 or EAEC strain O42, in addition to a range of isogenic mutant strains. E69 [LPS, non-motile, non-adherent, type three secretion system (TTSS) negative, signalling negative] or O42 [non-motile, non-adherent]. IL-8 and CCL20 protein secretion was measured. Bacterial surface structures were assessed by negative staining Transmission Electron Microscopy. The Fluorescent-actin staining test was carried out to determine bacterial adherence.

**Results:**

Previous studies have reported a balance between the host pro-inflammatory response and microbial suppression of this response. In our system an overall balance towards the host pro-inflammatory response is seen with the E69 WT and to a greater extent O42 WT, which is in fit with clinical symptoms. On removal of the external EPEC structures flagella, LPS, BFP, EspA and EspC; and EAEC flagella and AAF, the host inflammatory response is reduced. However, removal of E69 lymphostatin increases the host inflammatory response suggesting involvement in the bacterial mediated anti-inflammatory response.

**Conclusion:**

Epithelial responses were due to combinations of bacterial agonists, with host-bacterial contact a key determinant of these innate responses. Host epithelial recognition was offset by the microbe's ability to down-regulate the inflammatory response. Understanding the complexity of this host-microbial balance will contribute to improved vaccine design for infectious gastroenteritis.

## Introduction

Diarrhoeal disease is the second leading cause of infant mortality under the age of 5 worldwide with 1.5 million infant deaths each year, in addition to 1.1 million deaths in adults and infants over the age of 5 [1]. Common amongst the Diarrhoeagenic *Escherichia coli* strains is the ability to colonise the intestinal mucosa, evade host defences, multiply and cause host damage. One such strain, enteropathogenic *E. coli* (EPEC), is a human pathogen of the small intestine and is a significant cause of infantile diarrhoea [2]. The pathogenesis of EPEC involves three stages: *(I)* Initial adherence of bacteria to intestinal epithelial cells (IEC) in a characteristic pattern called “localised adherence” [3]. *(II)* Modulation of signal transduction *via* a type three secretion system (TTSS). EPEC injects a number of secreted effector proteins (Esp) directly into host cells, which can modulate host inflammation [2], [4]. If an inflammatory response ensues, it is due to the host pro-inflammatory response to EPEC outweighing the bacterial TTSS mediated anti-inflammatory response [4]. *(III)* Intimate adherence *via* an attaching and effacing (A/E) lesion [5], causing microvillus effacement that results in persistent watery diarrhoea, which ranges from non to weakly inflammatory [6].

Enteroaggregative *E. coli* (EAEC) is commonly associated with paediatric diarrhoea and malnutrition in developing countries; nevertheless EAEC is emerging as a significant diarrhoeal pathogen in adults, including HIV-positive patients and travellers. EAEC is a leading cause of food-borne outbreaks in the industrialized world and has been implicated in the development of post-infectious Irritable Bowel Syndrome [7]. There has been an important recent outbreak of severe haemolytic uraemia syndrome with unusually high mortality in German adults caused by a Shiga-toxin-producing EAEC (O104:H4) [8]. EAEC is defined by its distinctive “stacked-brick” aggregative adherence pattern [9]. The pathogenesis of EAEC involves three stages: *(I)* Adherence to the intestinal mucosa by aggregative adherence fimbriae (AAF) and adhesins. *(II)* Increased production of mucus that encrusts EAEC on the surface of enterocytes. *(III)* Release of toxins and elicitation of an inflammatory response and intestinal secretion. The AAF adhesin (AAF/II) also induces loss of epithelial integrity and delocalization of tight junction proteins, which may facilitate bacterial translocation to the submucosa [10]. Clinically, EAEC infection produces watery diarrhoea, occasionally with blood and mucus, and patients typically manifest intestinal inflammation, with production of pro-inflammatory cytokines such as IL-8 [7].

EPEC and EAEC possess several microbial-associated molecular patterns (MAMPs) recognized by host pattern recognition receptor (PRR) families, such as Toll-like receptors (TLR). Despite extensive studies the structures that specifically induce the IEC inflammatory response have not been unequivocally identified. Recent studies have highlighted the role of EPEC and EAEC flagellin monomer (FliC) alone in mediating IEC NF-κB and p38 MAPK activation leading to IL-8 production and gastroenteritis [6], [11]. In contrast to the well-established effect of FliC, the effect of bacterial adherence to IEC innate response(s) remains unclear. As past findings suggest IL-8 production requires the TTSS and intimate adherence in the case of EPEC [12] and the bacterial aggregative adherence plasmid (pAA) in the case of EAEC [13]. In the present study the aim was to characterise and identify, which bacterial motifs contribute to the production of IL-8 and CCL20. We tested the hypothesis that bacterial adherence contributes to IEC antimicrobial innate immunity during EPEC/EAEC infection. For this purpose an array of isogenic mutants were employed in co-culture studies; our data suggests that ‘epithelial contact’ is indeed a key determinant defining IEC-pathogen crosstalk.

## Results

### Bacterial morphology by negative staining transmission electron microscopy (TEM)

**Figure 1:**
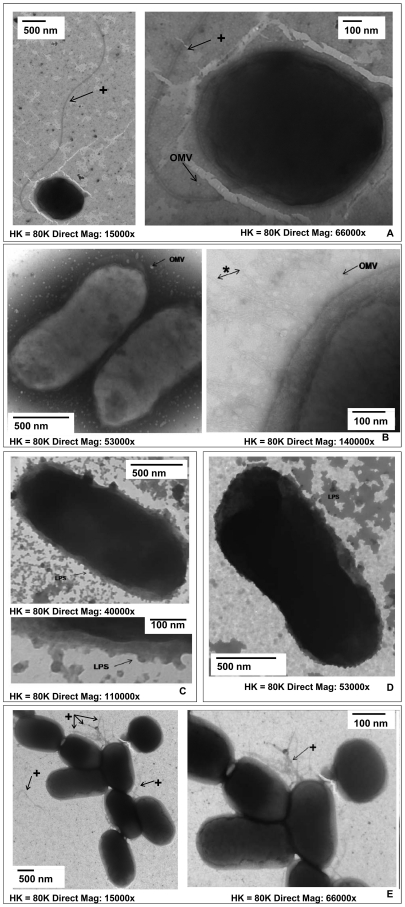
Structure of pathogenic EPEC E69. WT strain (a); LPS rough (b) and smooth mutant strains (c); flagella mutants E69 *ΔfliC* (d) and *fliC^+^* (e) as observed by negative staining TEM. Magnification range was between 8800- 66000x. [OMV - outer membrane vesicles; LPS - O-polysaccharide chains of LPS; * - fimbriae; + - flagella].

To gain a greater insight into bacterial-host interactions we determined the presence of microbial structural components known to interact with host PPRs, using negative staining followed by TEM. The E69 WT was found to express a monotrichous flagellum as well as outer membrane vesicles (OMV) (Figure 1a). OMVs are spherical bi-layer vesicles, which are constantly discharged from the bacterial surface during normal growth and can be considered virulence factors [14]. The rough LPS mutant expressed fimbriae and OMVs and lacked O-polysaccharide chains (Figure 1b). The E69 LPS smooth mutant strain expressed O-polysaccharide chains of LPS, which appeared to bleb off the surface of the bacterium in a different manner to that of OMVs (Figure 1c) and was not observed for the WT strain (Figure 1a). The E69 flagella mutant E69 Δ*fliC* did not express any flagella (Figure 1d). In fact, this strain had a strong resemblance to the E69 smooth mutant strain, in that it too had O-polysaccharide chains of LPS, which could be seen to bleb off the bacterial surface. The E69 flagella enhanced strain (*fliC* ^+^) was found to express lophotrichous flagella and OMVs (Figure 1e).

**Figure 2:**
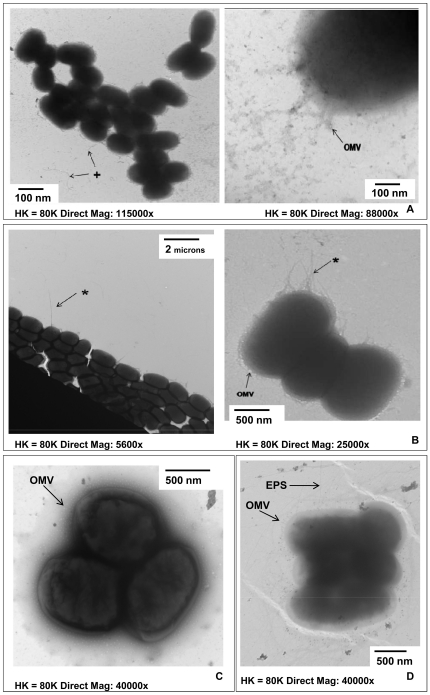
Structure of pathogenic EAEC O42. WT strain (a) and O42 *ΔfliC* (b) mutant; O42 aggregative adherence fimbriae mutants O42 *ΔAafB* (c) and O42 *ΔAAF/II* (d) as observed by negative staining TEM. Magnification range was between 8800- 66000x. [OMV - outer membrane vesicles; LPS - O-polysaccharide chains of LPS; * - fimbriae; + - flagella; EPS - exopolysaccharide].

The EAEC WT serotype O42 expressed OMVs and flagella, and formed colonies resembling a stacked brick formation (Figure 2a). The O42 Δ*fliC* mutant strain did not express flagella, but did express fimbriae and OMVs, forming colonies in the characteristic stacked brick formation (Figure 2b). The aggregative adherence fimbrial adhesin mutant *AafB* expressed OMVs only (Figure 2c). Another aggregative adherence fimbria mutant AAF/II expressed a rope like exopolysaccharide and OMVs, but did not express fimbriae (Figure 2d). Collectively, the majority of strains investigated expressed multiple microbial structural components known to interact with host PPRs inducing antimicrobial responses. We therefore went on to investigate the effects of several of the surface structures on the host response.

### Recognition of EPEC and EAEC flagella contributes to the IEC innate immune response

#### HEp-2 cells respond to apical stimulation with flagellated E69

**Figure 3:**
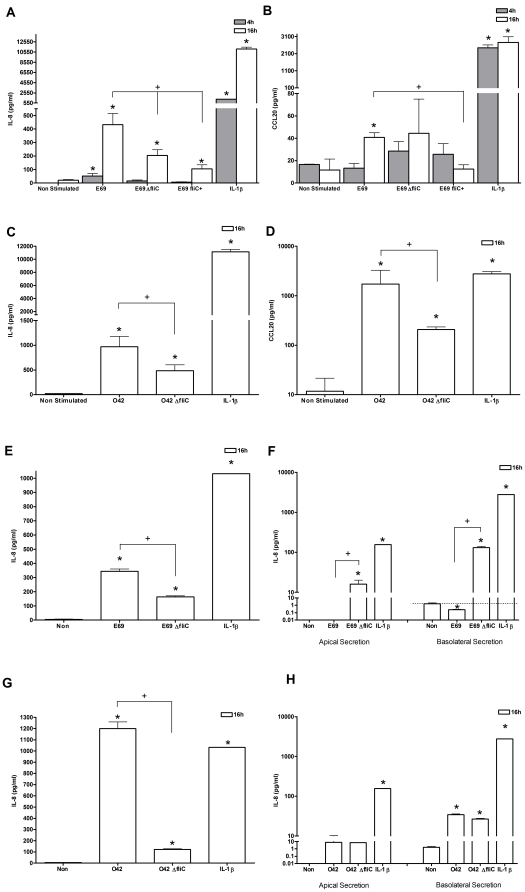
HEp-2 and Caco-2 cells respond to apical stimulation with flagellated EPEC and EAEC; however, Caco-2 cells are un-responsive to basolateral stimulation with flagellated EPEC. Cells were either co-cultured with E69, the E69 flagella mutant Δ*fliC* strain, the E69 flagella enhanced strain *fliC^+^*, O42 and O42 Δ*fliC* (MOI = 60), or stimulated with IL-1β (10 ng/ml) as a positive control. At 4 or 16 h post-infection with E69 or O42, HEp-2 cell and bacterial co-culture supernatants were harvested and IL-8 (a & c) or CCL20 (b & d) evaluated by ELISA. Caco-2 cells were stimulated apically for 16 h and IL-8 protein evaluated (e & g). Caco-2 cells were also stimulated basolaterally for 16 h, apical and basolateral IL-8 secretion was evaluated (f & h). Data shown is mean (+SD,*/+ P≤0.05) induction of three independent experiments.

As E69 flagellin the subunit of flagella has been reported to induce IL-8 secretion by IECs [12] we determined the response to flagella in our model system. Initially, we investigated the effects of apical stimulation with flagellated bacteria, as it is likely that this will be the first interaction between E69 and intact “healthy” mucosa. Viable E69 WT and E69 isogenic flagella enhanced (*fliC* ^+^) and non flagellated (Δ*fliC*) mutants were investigated. At 4 h, it was only E69 that induced any significant secretion of IL-8 (Figure 3a). None of the strains induced any significant CCL20 protein in HEp-2 cells at 4 h (Figure 3b). At 16 h the E69 WT and the two flagella mutants induced a significant increase in IL-8 protein (Figure 3a). Only the WT strain E69 induced significant production of CCL20 protein at this time point (Figure 3b). A statistically significant reduction in IL-8 expression between the WT strain and the E69 Δ*fliC* mutant was observed, indicating that flagella may play a role in apical HEp-2 cell recognition and immune response. There was no significant difference in the induction of CCL20 between the WT strain and the E69 Δ*fliC* flagella deficient strain (Figure 3b). The flagella enhanced strain E69 *fliC^+^* showed a more significant reduction in IL-8 and CCL20 in comparison to the WT than the E69 Δ*fliC* flagella deficient strain (Figure 3a & b). It has previously been suggested that EPEC has the ability to secrete proteins *via* its flagella apparatus, which may modulate IEC host responses [15].

#### HEp-2 cells respond to apical stimulation with flagellated O42

We next investigated the response of HEp-2 cells to apical stimulation with the EAEC WT strain O42 and its isogenic flagella mutant O42 Δ*fliC* was investigated. At 16 h the O42 WT and the flagella mutant both induced a significant amount of IL-8 and CCL20 protein (Figure 3c & d). Indeed, stimulation with the O42 WT induced significantly higher levels of the pro-inflammatory cytokines in comparison to stimulation of HEp-2 cells with the E69 WT (Figure 3a & b). The flagella negative mutant showed reduced ability to induce IL-8 and CCL20 protein when compared to the WT strain, implying that flagella are involved in apical HEp-2 cell recognition and response to O42.

#### Caco-2 cells respond to apical stimulation with flagellated E69 and O42

We examined the response of Caco-2 cells to apical stimulation with the EPEC WT strain E69, EAEC WT strain O42 and their respective flagella negative mutant strains E69 ΔfliC and O42 ΔfliC. At 16 h the E69 and O42 WTs and the flagella mutants induced a significant increase in IL-8 protein, although the flagella negative mutants showed a reduced ability to induce IL-8 protein when compared to the WT strain (Figure 3e & g). The reduction seen with the flagella mutants suggest that flagella do play a role in the apical Caco-2 cell response to EPEC and EAEC, as has been previously reported [12], [16].

#### Caco-2 cells are un-responsive to basolateral stimulation with flagellated E69

As TLR5, the receptor for flagellin, is reported to be located on the basolateral membrane [17], the response of Caco-2 cells to basolateral stimulation with the EPEC WT strain E69 and the flagella Δ*fliC* mutant strain was investigated. Supernatant was collected from the apical and basolateral compartments. Upon basolateral inoculation for 16 h, no IL-8 protein was secreted into the apical compartment by control and E69 WT infected cells (Figure 3f). A modest amount of apical IL-8 secretion was seen with the flagella negative mutant. Minimal IL-8 was detected in the basolateral compartment of control cells. During infection with the WT strain, IL-8 secretion into the basolateral chamber was found to be below the levels measured in uninfected control cells. In comparison, a marked increase in IL-8 protein was observed on stimulation with the flagella mutant. Thus, the aflagellated strain induced a statistically significant increase in IL-8 secretion in comparison to the WT strain both apically and basolaterally (Figure 3f). These data suggest a role of flagella in the inhibition of the host response to basolateral inoculation with EPEC WT E69.

#### Caco-2 cells are responsive to basolateral stimulation with flagellated O42

The response of Caco-2 cells to basolateral stimulation with the EAEC WT strain O42 and the flagella *ΔfliC* mutant strain was investigated. Supernatant was collected from the apical and basolateral compartments. Upon basolateral inoculation for 16 h, a modest amount of apical IL-8 secretion was seen with the O42 WT and the flagella negative mutant (Figure 3 h). Minimal IL-8 was detected in the basolateral compartment of control cells with an increase in IL-8 secretion into the basolateral chamber during infection, with the WT strain and the flagella mutant, showing no significant difference (Figure 3 h). These data suggest that flagella are not involved in the host response to basolateral inoculation with EAEC WT O42.

Collectively, these data do indicate a role for flagella in apically induced immunity. However, as the IEC responses were not completely ablated on apical stimulation with the flagella mutants in comparison to the WT, we hypothesised that in addition to flagella other bacterial factors may be involved in IEC recognition.

### Recognition of the O-polysaccharide chains of EPEC lipopolysaccharide (LPS) contributes to the IEC innate immune response

LPS is the dominant component of the outer membrane of gram-negative bacteria and is released when bacteria multiply and die [18]. Therefore, the involvement of LPS to host recognition was investigated. LPS consists of a lipid A moiety, a core polysaccharide and O-polysaccharide chains of variable lengths. Colony morphology is indicative of O-glycosylation status. Smooth colony forming strains express the complete core with varying chain lengths, whereas rough colony forming mutants lack the O-polysaccharide chains [19]. Despite extensive studies the LPS structures (i.e. core or polysaccharide chains) that induce IEC inflammatory responses have not been unequivocally identified. In this series of experiments, the involvement of EPEC WT E69 LPS was investigated with the use of isogenic rough and smooth LPS mutant strains. The TLR4/MD-2/CD14 receptor complex recognises LPS. Due to the conflicting reports as to whether Caco-2 cells express biologically active TLR4, MD-2 or CD14 [20], [21] the host innate immune response to EPEC LPS in HEp-2 cells was established.

**Figure 4:**
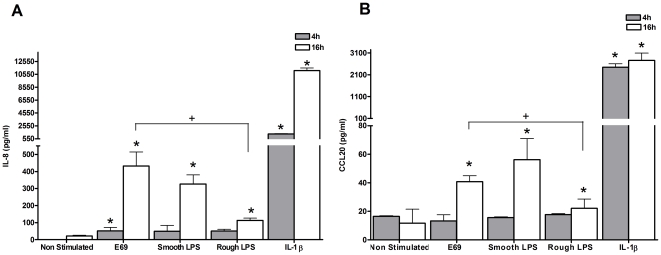
The O-antigen of LPS contributes to the IEC innate immune responses. HEp-2 cells were either co-cultured with E69, E69 smooth LPS or E69 rough LPS (MOI = 60), or stimulated with IL-1β (10 ng/ml) as a positive control. 4 or 16 h post-infection cell and bacterial co-culture supernatants were harvested and IL-8 (a) and CCL20 (b) protein levels evaluated by ELISA. Data shown is mean (+SD,*/+ P≤0.05) induction of three independent experiments.

On exposure to WT E69, but not rough/smooth LPS mutants, a minimal increase in IL-8 protein secretion 4 h post-infection was noted (Figure 4a). However, induction in IL-8 and CCL20 protein in response to all strains was observed 16 h post-bacterial exposure (Figure 4a & b). There was a modest, but not significant increase in CCL20 expression between the WT and smooth LPS isogenic mutant strains (Figure 4b). The rough LPS strain exhibited reduction in its ability to induce both IL-8 and CCL20 protein expression in comparison to the WT (Figure 4a & b). These data are indicative of a role for O-polysaccharide chains in LPS–induced immunity.

### Significant contribution of adherence to IEC innate immune response

Once again, the inflammatory response was not completely ablated upon infection with the LPS mutants, in comparison to the WT bacteria; thus in addition to flagella and LPS other bacterial factors are involved in IEC responses. We hypothesised that pro-inflammatory responses may result from the attachment of EPEC and EAEC to host cells. This is particularly relevant, given that aggregative adherence and intimate attachment to IECs are hallmarks of EAEC and EPEC infection respectively. In the following series of experiments the contribution of adherence to HEp-2 cell responses of EPEC WT strain E69 was investigated.

#### Localised adherence of EPEC

The first stage of EPEC colonisation is a characteristic pattern of adherence called “localised adherence”. Several bacterial surface-organelles and secreted products have been implicated in this adherence; such as the bundle forming pilus (BFP), type I fimbriae and EPEC secreted protein A (EspA) [22]. BFP is a type IV fimbriae encoded on a large plasmid called the EPEC adherence factor plasmid (EAF) [9]. The role of BFP was studied with isogenic mutant *E. coli* strains JPN15 (lacks the EAF plasmid) and 31-6-1(1) (with a TnphoA insertion - an inactivation mutation in the virulence plasmid-encoded *bfpA* gene). Type I fimbriae adherence was inhibited by the addition of mannose to infections of WT E69 [23]. EspA filaments of the TTSS needle complex adhere EPEC to the host cell [24]. The role of EspA was investigated by using a *ΔespA* isogenic mutant.

**Figure 5:**
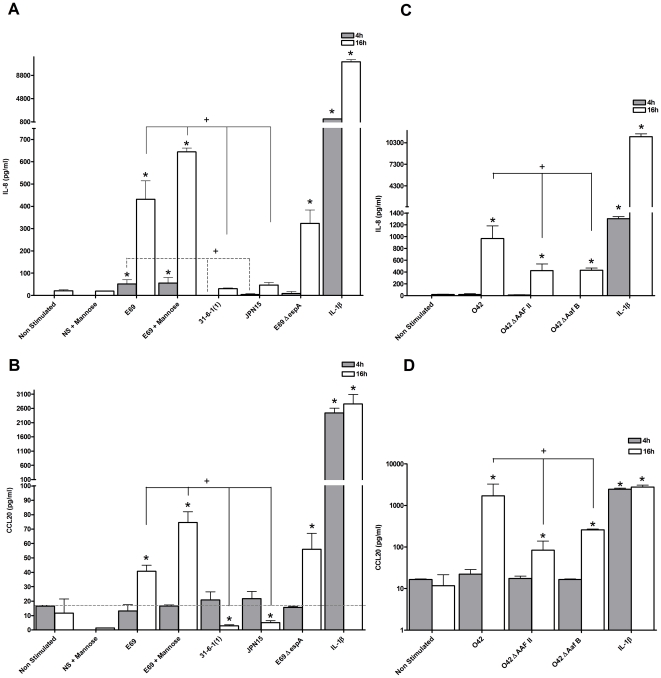
Adherence significantly contributes to innate recognition of EPEC and EAEC. HEp-2 cells were either co-cultured with E69, E69 plus mannose (5 µg/ml), E69 31-6-1(1), E69 JPN15, E69 *ΔespA;* O42, O42 *ΔAAF/II*, O42 *ΔAafB* (MOI = 60), or stimulated with IL-1β (10 ng/ml) as a positive control. At 4 or 16 h post-infection with E69 or O42 co-culture supernatants were harvested and IL-8 (a & c) and CCL20 (b & d) evaluated by ELISA. Data shown is mean (+SD, */+P≤0.05) induction of three independent experiments.

At 4 h the E69 WT and the WT plus mannose caused a modest induction in IL-8 protein (Figure 5a). Strains JPN15 and 31-6-1(1) showed no expression of IL-8 protein. None of the strains investigated induced any significant change in expression of CCL20 protein at 4 h (Figure 5b). At 16 h the E69 WT, the WT plus mannose and the EspA mutant induced a marked increase in IL-8 and CCL20 protein (Figure 5a & b). At 16 h in comparison to the WT, JPN15 and 31-6-1(1) showed a statistically significant reduction in expression of IL-8 and CCL20 protein (Figure 5a & b). The levels of CCL20 protein were found to be even lower than the ‘constitutive’ expression noted in uninfected control cells. Suggesting that recognition of BFP contributes to the host pro-inflammatory response and the inflammatory response is not stimulated in the absence of BFP. Furthermore, as the levels are below the constitutive expression the balance between host and microbe has been tipped in favour of bacterial suppression. E69 plus mannose induced the most statistically significant increase in IL-8 and CCL20 protein amongst the strains tested (Figure 5a & b). There was no significant difference in IL-8 and CCL20 protein expression between the EspA mutant and E69 WT (Figure 5a & b). Collectively these data implicate adherence of E69 *via* BFP as a strong determinant of IL-8 and CCL20 expression.

#### Aggregative adherence of EAEC

EAEC has a distinctive aggregative adherence, with a ‘stacked brick-like’ pattern distinguishable from that manifested by EPEC [9] as seen in (Figure 2b). EAEC adherence requires expression of aggregative adherence fimbriae (AAFs). EAEC prototype strain O42 expresses the AAF/II allele, encoded on plasmid pAA2. The AAF/II fimbriae is 5 nm in diameter and mediates adhesion to HEp-2 cells and colonic explants [25]. A fimbriae negative strain (O42 *Δ*AAF/II isogenic mutant) and adhesin negative strain (O42 *ΔAafB* isogenic mutant) were utilised. At 4 h there was no statistically significant induction of IL-8 and CCL20 protein expression (Figure 5c & d). However, at 16 h all strains induced a statistically significant secretion of IL-8 and CCL20 (Figure 5c & d); both mutant strains reduced the response in comparison to the WT strain. This suggests that adherence of O42 *via* AAF and the AAFB adhesin does contribute to IL-8 and CCL20 expression.

### Recognition of structural and effector components of the EPEC TTSS contributes to the host innate immune response

Our data illustrates that the first stage of pathogenesis of EPEC and EAEC, localised adherence to IECs (EPEC *via* BFP, EAEC *via* AAF/II), is key to the host pro-inflammatory response. Interestingly however, the second stage of EPEC infection is characterised by the TTSS, in a contact dependant manner, injecting effector proteins into IECs, which can result in an anti-inflammatory response [4]. Therefore, it was of interest to investigate the effect of bacterial contact *via* the TTSS and its effectors on the host pro-inflammatory response in our system.

Briefly, in the second stage of EPEC infection EscF binds to EspA, the structural needle protein, and forms a 0.7 µm long hollow extension of the TTSS needle complex. This is made up of polymorphic EspA filaments, through which the EspB and EspD passes before inserting into the host plasma membrane to form a translocation-pore, thus allowing translocation of LEE encoded effector proteins into the host cell cytosol to interfere with signalling processes [26]. EscN a functionally unique ATPase provides an inner-membrane recognition gate for the TTSS chaperon-virulence effector complexes as well as a source of energy for their subsequent secretion [27].

At this stage what remains unclear is the contribution of the secreted effector proteins *versus* that of the syringe apparatus itself in eliciting an immune response, as there are several contradictory reports in the literature. For example, Sharma *et al.* indicate that a functional TTSS is required for an anti-inflammatory response [4]. In contrast, other authors have reported that the TTSS is necessary for the activation of MAPK pathways and IL-8 production, while a minimal role for the EPEC TTSS has also been reported [28].

**Figure 6:**
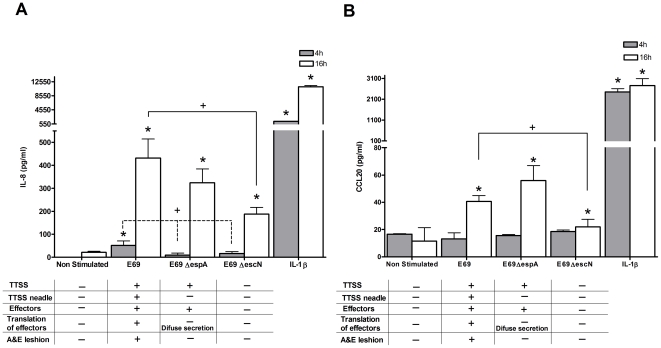
Structural components of the TTSS affect the IEC innate immune response. HEp-2 cells were either co-cultured with E69, E69 Δ*escN*, E69 Δ*espA* (MOI = 60), or stimulated with IL-1β (10 ng/ml) as a positive control. At 4 or 16 h post-infection co-culture supernatants were harvested and IL-8 (a) and CCL20 (b) were evaluated by ELISA. Data shown is mean (+SD, */+P≤0.02) induction of three independent experiments.

To investigate the potential role of the structural components of the TTSS in IEC immune response(s) to EPEC, the isogenic mutant strains Δ*espA* and Δ*escN* were used. The Δ*espA* strain does not express the needle portion of the TTSS, but does diffusely secrete effector proteins. The Δ*escN* strain does not express a TTSS nor does it secrete effector proteins. At 4 h, only E69 induced a modest, but significant expression of IL-8 protein (Figure 6a). A reduction was noted during infection with both TTSS mutant strains, with no strains modulating CCL20 production at this time (Figure 6b). At 16 h all strains induced a significant expression of IL-8 and CCL20 protein (Figure 6a & b). Stimulation with E69 Δ*espA* gave a greater increase in CCL20 when compared to the WT albeit not statistically significant. The Δ*escN* strain caused significant reduction in IL-8 and CCL20 expression when compared to the WT strain (Figure 6a & b). Thus, in the model system employed here, recognition of structural components of the TTSS and more significantly, the effector proteins appear to be involved in the pro-inflammatory responses to E69.

### EPEC effector proteins can both contribute to or modulate IEC innate immune responses

**Figure 7:**
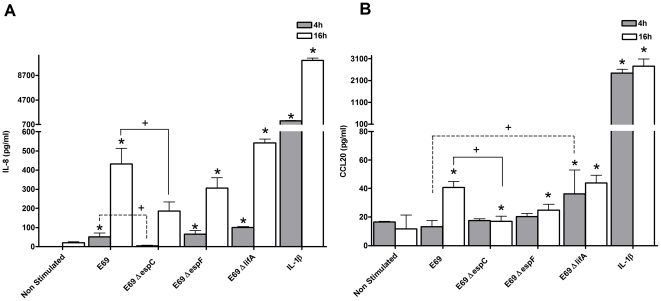
EPEC effector proteins can either induce or modulate IEC innate immune responses. HEp-2 cells were either co-cultured with E69, E69 Δ*espC*, E69 Δ*espF*, E69 Δ*lifA* (MOI = 60), or stimulated with IL-1β (10 ng/ml) as a positive control. At 4 or 16 h post-infection co-culture supernatants were harvested and IL-8 (a) and CCL20 (b) were evaluated by ELISA. Data shown is mean (+SD, */+P≤0.05) induction of three independent experiments.

The contribution of the secreted effector proteins in eliciting an immune response was investigated further using Δ*espC*, Δ*espF* and *ΔlifA* isogenic mutant strains. EspC causes cytotoxic effects including cytoskeletal damage to IECs [29]. EspF functions in immune evasion [30]. The *lifA* gene encodes for a toxin, lymphostatin, a non-TTSS-secreted protein, which inhibits peripheral blood and also human and murine GI lymphocyte proliferation plus cytokine production [31]. Upon inoculation with the Δ*espC* strain for 4 h there was a statistically significant reduction in IL-8 protein in comparison to the WT (Figure 7a). No significant induction of CCL20 was seen at this time. At 16 h there was a statistically significant reduction in both IL-8 and CCL20 protein with the Δ*espC* strain in comparison to the WT (Figure 7a & b), suggesting involvement of EspC in host recognition. The lymphostatin negative strain induced significant IL-8 and CCL20 secretion at 4 h (Figure 7a & b). In comparison to the WT, CCL20 secretion was significantly enhanced with the Δ*lifA* strain (Figure 7b). Suggesting that lymphostatin may exert an inhibitory effect on epithelial responses. At 16 h however, the IL-8 and CCL20 levels induced by the *ΔlifA* mutant strain were similar to the WT strain. The Δ*espF* mutant induced IL-8 protein secretion at 4 and 16 h (Figure 7a) and CCL20 secretion at 16 h (Figure 7b). The levels of induction of IL-8 and CCL20 at 16 h in comparison to the WT were reduced, but not significantly, indicating a minimal role of EspF in modulating IL-8 and CCL20 responses in this system.

### EPEC BFP, TTSS and lymphostatin implicated in the balance between host induction and EPEC inhibition of IL-8 mRNA

**Figure 8:**
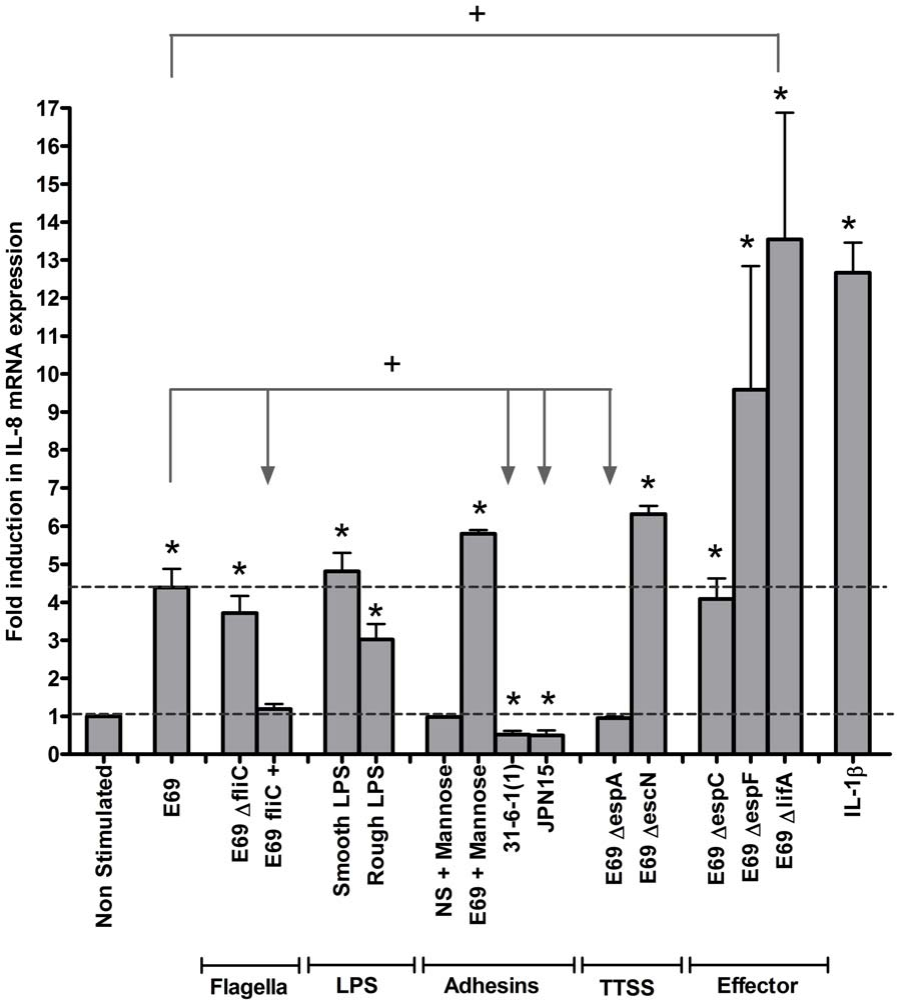
EPEC BFP, TTSS and lymphostatin implicated in the balance between host induction and EPEC inhibition of IL-8 mRNA. IL-8 gene expression of HEp-2 cells 4 h post-infection was determined and normalised to GAPDH. Variations in mRNA levels are expressed as fold induction compared to the uninfected control cells. Data shown is mean (+SD,*/+ P≤0.05) induction of three independent experiments. A representative gel is included (see Figure S1).

During the second stage of infection EPEC modulates signal transduction *via* the TTSS [2], [4], if an inflammatory response ensues it is due to the host pro-inflammatory response to EPEC outweighing the bacterial mediated anti-inflammatory response [4]. To investigate the potential of EPEC to modulate IEC immune responses in our model system, IL-8 mRNA expression of HEp-2 cells was determined 4 h post inoculation. At 4 h the E69 WT induced a significant up-regulation of IL-8 mRNA expression (Figure 8 & Figure S1), suggesting an overall balance towards the host pro-inflammatory response. On exposure to the flagella mutant (Δ*fliC*) and the rough/smooth LPS mutants significant IL-8 mRNA expression was observed however, there was no significant difference in expression compared to the WT. There was no induction of IL-8 mRNA expression with the flagella enhanced strain (*fliC* ^+^) and the TTSS mutant *ΔespA*, thus showing significant reduction in expression compared to the WT and suggesting that recognition of EspA contributes to the host pro-inflammatory response. In addition, flagella may be involved in suppression of the host response. E69 WT incubated in the presence of mannose induced a significant increase in IL-8 mRNA, not seen with stimulation of mannose alone. Upon infection with the BFP mutant strains JPN15 and 31-6-1(1), levels of IL-8 mRNA were found to be even lower than the ‘constitutive’ expression noted in uninfected control cells and were significantly reduced in comparison to the WT strain. Therefore, on removal of the external bacterial structure BFP the host inflammatory response is not stimulated and a bacterial mediated anti-inflammatory response then outweighs the host pro-inflammatory response to EPEC. There was significant induction of IL-8 mRNA expression with the Δ*escN*, Δ*espC* and Δ*espF* strains, but no significant difference in expression compared to the WT was observed. The lymphostatin negative strain induced statistically significant expression of IL-8 mRNA compared to the WT; suggesting that lymphostatin inhibits IL-8 mRNA expression. Overall our data suggest that there is a delicate balance between the host pro-inflammatory response and EPEC suppression of this response.

### Contribution of the EPEC intimate adherence to IEC immune response

As our data demonstrates that localised adherence to IECs is key determinant of the host pro-inflammatory response to EPEC it was important to investigate the third stage of EPEC infection characterised by intimate adherence to the mucosal membrane forming an attaching and effacing lesion. In this series of experiments the intimate adherence status of the EPEC strains were investigated using the FAS test. Ultra structural studies show the accumulation of cytoskeletal actin, beneath intimately attached bacteria. Knutton *et al.* developed a specific fluorescent-actin staining (FAS) test, which is diagnostic for the AE adherence property [32]. The nucleation of actin by bacteria to form filamentous or F-actin, which is stained by FITC conjugated phalloidin, allows the bacteria to be divided into fluorescent actin staining (FAS) positive (intimately-adherent) or FAS negative bacteria (non-adherent).

The control, uninfected HEp-2 cells at 16 h showed a typical negative FAS test, with actin fluorescence localised at the cell periphery (Figure S2). Upon infection with E69 WT the FAS test was positive, with fluorescence localizing at the cell periphery in addition to intense spots of actin fluorescence, which in comparison with the complementary phase-contrast image, correspond in size and position with adherent bacteria (Figure S2). Thus, the WT strain behaved as previously described [32]. The EPEC E69 flagella negative mutant (Δ*fliC*) and flagella enhanced strain (*fliC^+^*) were both FAS test positive, as were the EPEC E69 LPS smooth and rough mutant strains (data not shown). This demonstrates that the mutant strains are able to form AE lesions in a manner similar to the WT strain, indicating that these mutations do not alter the strain phenotype or their adhesion status and therefore, any reduction seen with the mutant strains is not due to alteration in intimate adhesion status. The EPEC E69 bundle forming pilus mutant 31-6-1(1), which has a TnphoA in *bfpA* no longer conferring localised adherence, was FAS test negative, with actin fluorescence localised at the cell periphery (data not shown). These data would suggest that initial localised adherence may influence intimate adherence and the formation of AE lesions. However, the EPEC E69 mutant JPN15 that lacks the EAF plasmid on which BFP is encoded [33], preventing localised adherence *via* BFP, was weakly FAS test positive, showing characteristic spots of actin fluorescence corresponding in size and position with adherent bacteria (data not shown). This suggests that the JPN15 strain utilises an alternative to BFP for initial localised adherence prior to forming an AE lesions, such as type I fimbriae, EspA or flagella [22]. The TTSS mutant strains Δ*espA* and Δ*escN* were confirmed as FAS test negative (data not shown).

**Figure 9:**
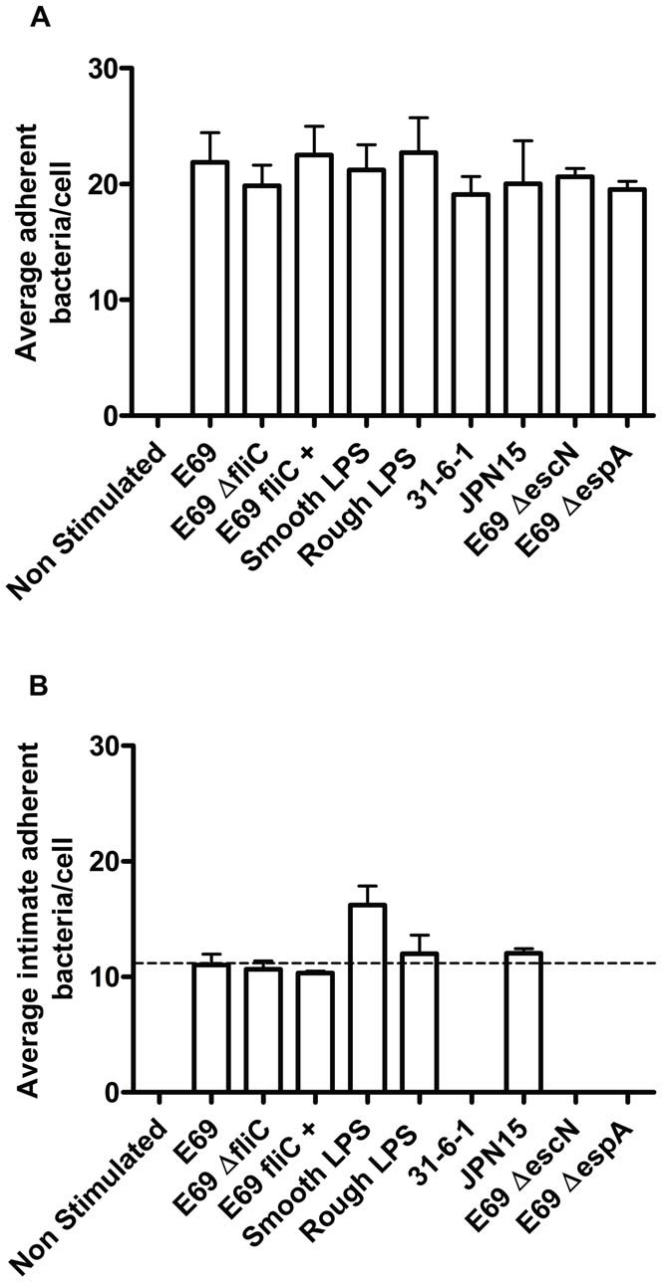
Quantification of localised (a) and intimate (b) adherent E69 at 16 h. Following the fluorescent-actin staining test, complementarity of bacterial location and actin fluorescence was confirmed by simultaneously recording phase-contrast and fluorescence images. The micrographs were analysed with ImageJ software (NIH) to quantify the number of adherent bacteria per cell. Data shown as mean (+SD) of three independent experiments.

Upon quantification of the number of localised or intimate adherent bacteria using ImageJ software, we found similar levels of adherent bacteria for all strains, with an average of twenty adherent bacteria per cell from an initial inoculate of sixty per cell (Figure 9a). This shows that any reduction in the inflammatory response observed with the mutant strains in comparison to the WT was not due to alterations in the levels of infectivity for the strains, but due to removal of the bacterial surface structures themselves. For the majority of strains an average of ten intimately adherent bacteria per cell was observed. Thus, approximately fifty percent of the adherent bacteria were intimately adhered (Figure 9b), which may explain the overall balance to a pro-inflammatory response; as only half of the adherent bacteria would have the ability to suppress the host response. No intimate adherence was seen with the BFP mutant strain 31-6-1(1), or the E69 ΔespA and ΔescN TTSS mutants (Figure 9b). Interestingly, an increased percentage of intimately adhered bacteria were observed with the smooth LPS strain (from 50 to 75%), which may account for the differences observed in IL-8 and CCL20 expression between the WT and smooth LPS isogenic mutant strain. Also, while not statistically different from the WT, a 25% difference in expression of IL-8 and CCL20 is observed with the smooth LPS strain (Figure 4). Thus we found a correlation between the number of intimately adhered bacteria and the level of the host response observed.

## Discussion

This study aimed to characterise the bacterial motifs on EPEC and EAEC that induce epithelial IL-8 and CCL20 responses [34], [35]. We investigated the contribution of “native” flagella rather than using flagellin monomers, finding a role in apical HEp-2 and Caco-2 cell recognition and immune response. This contrasts with reports of basolateral responses to the flagellin subunit [17]. Others have noted differential host responses to flagellated *versus* aflagellated bacteria and intact flagella *versus* “monomeric” flagellin protein [36], [37]. The E69 *ΔfliC* mutant induced greater IL-8 secretion than wild type E69 basolaterally, suggesting that flagella inhibit host responses at this site, possibly by their secretion of inhibitory effector proteins at the basolateral surface [15]. This is an active process requiring the presence of viable bacteria, which may suppress host responses after epithelial barrier disruption. In support of this, the flagella enhanced strain E69 *fliC^+^* induced greater reduction in IL-8 and CCL20 than the E69 Δ*fliC* flagella deficient strain. There is currently no flagella enhanced EAEC O42 strain to determine whether this is a generalised phenomenon. We investigated the role of LPS in apical response, which was maintained in flagella mutants. Our data demonstrated a role for O-polysaccharide chains in LPS–induced immunity. Variability in O-antigen similarly affects both responses and signalling pathways in macrophages [19]. O-antigen also contributes to localised adherence of EPEC [38], implicating a potential role for attachment in the host response. We found that the IEC immune response was not completely ablated with the rough mutant, suggesting that LPS is not the only factor likely to be involved in apical response to E69.

We hypothesised that pro-inflammatory responses may result from the attachment of EPEC and EAEC to host cells. The striking reduction of IL-8 and CCL20 production in response to BFP mutants identifies BFP as a major determinant of the host inflammatory response. The BFP mutant JPN15 was able to form a TTSS and induce intimate adherence, but inhibited NF-κB responses. Thus, without host recognition of BFP the balance is shifted towards an anti-inflammatory response. In support of this, JPN15 can adhere to human intestinal mucosa and produce the AE lesion [32], [39], but does not induce neutrophils to cross the epithelium [40]. Bacterial suppression was also seen with the 31-6-1(1) mutant, but this strain was unable to form intimate adherence. Suppression seen with this strain is potentially TTSS independent, and we found that the EPEC WT E69 down-regulated the host response *via* secretion of lymphostatin.

E69 in the presence of mannose induced the greatest increase in IL-8 and CCL20 protein. Although mannose inhibits adherence *via* type I fimbriae, E69 can still bind to the epithelia *via* a variety of additional mechanisms such as BFP, flagella and EspA. The augmentation with mannose suggests that its binding to E69 may act as a MAMP.

There have been conflicting reports of the role of the TTSS in host pro- and anti-inflammatory responses [2], [4], [28]. Our data indicate that both the structural component of the TTSS and effector proteins are involved in the pro-inflammatory responses to E69. The secreted effector protein EspC was involved in host recognition, while EspF did not modulate IL-8 and CCL20 responses. The Δ*lifA* strain induced increased IL-8 mRNA expression and CCL20 secretion compared to that of the WT strain. As lymphostatin inhibits peripheral blood and mucosal lymphocyte proliferation and cytokine synthesis [31], it may similarly inhibit epithelial responses. The *lifA* gene of atypical EPEC is indeed strongly associated with diarrhoea, supporting its role in virulence [41].

We noted a degree of variability in IL-8 mRNA correlation with protein secretion, as has been previously reported and is likely due to post-translational or transcriptional control mechanisms [42]. Despite the variations, similar trends in mRNA and protein suppression *via* bacterial-epithelial contact (with BFP, flagella or EspA) were found, indicating, by whatever mechanism, the suppression has affected these control mechanisms. IECs thus recognise a combination of EPEC E69 PRR agonists. Host-bacterial contact is significant, as are flagella, LPS, EspA, EspC and the TTSS. In different model systems, EPEC flagella with either EscN [12] or intimin [43] augment host response. EPEC intimate adherence also modulates TLR5 localization and host signalling [43], further supporting the importance of host-bacterial contact.

The EAEC WT O42 also induced strong expression of IL-8 and CCL20; indeed greater than the E69 WT, consistent with its greater *in-vivo* propensity to induce inflammatory diarrhoea [6], [7]. Although EPEC can suppress the immune response *via* the TTSS, EAEC does not do this, but induces an inflammatory response likely to subvert the epithelial barrier [44]. These findings provide insight into the enhanced severity of disease caused by Shiga toxin producing EAEC (O104:H4) during the recent German epidemic [8], in comparison to classic EHEC infection, as systemic dissemination of Shiga toxin is likely to be promoted by the increased ability of EAEC to induce an epithelial pro-inflammatory and chemokine response.

Our data suggest that flagella are involved in apical HEp-2 and Caco-2 cell recognition and response to O42, although the epithelial response to the O42 Δ*fliC* mutant was not completely eliminated, and other factors are likely involved. We hypothesised that pro-inflammatory responses may result from attachment of EAEC to host cells in their distinctive aggregative adherence, with a ‘stacked brick-like’ pattern [9], which is key to pathogenesis. Our data indicated that adherence *via* AAF indeed contributes to IL-8 and CCL20 expression. However, the response is not completely abolished without adherence and recognition of flagella also played a role in host recognition of EAEC O42. This extends previous studies to show that EAEC has a heterogeneous ability to modulate IL-8 [13], [45].

Our data provide findings distinct from previous studies using isolated bacterial components such as flagellin monomer. For both EPEC E69 and EAEC O42, isogenic mutants depleted in various structures induced, reduced responses in comparison to the WT. These were however, rarely fully ablated, suggesting a response to more than one component. These data concord with previous findings that factors other than flagellin monomers, including EPEC TTSS and intimate adhesion [12] and EAEC bacterial pAA plasmid [13], impact on the host response. Many non-pathogenic bacteria express flagella and readily release flagellin monomers, and the immune system may thus encounter flagellin more frequently than other bacterial products such as LPS [46]. It would be undesirable for the host to mount a response solely on the presence of flagellin, more likely derived from gut commensals than pathogens. Here, we show that the host response to the whole flagellated bacteria differs to that of the flagellin monomer and the overall innate immune response is due to the recognition of groups of external structures. Hedlund *et al.* also suggest the host ‘sees’ microbial products not as purified molecules, but as complexes [47]. TLRs do not function in isolation, but form multi-receptor complexes with other PRRs in membrane lipid rafts. Ligand-induced PRR oligomerization modifies the arrangement of TIR domains, altering the binding specificity required for recruitment of appropriate adaptors, allowing combinational diversity in PRR signal transduction pathways [48]. The PRR responsible for detection of type I fimbriae, which bind to mannose, is TLR4 [49]. It would be of interest to determine the PRRs responsible for the recognition of BFP, EscN and the TTSS. The fact that the host responds to combinations of virulence factors has important implications, as failure to develop effective vaccines for complex pathogens may relate to the fact that vaccines to date are mainly to one virulence factor, while simultaneous disruption of multiple virulence factors may be required [50]. Our data support this hypothesis.

Adherence was a strong determinant in induction of the IL-8 and CCL20 response to E69 and O42. It may be that such contact dependence is due to the requirement for docking to the cell, allowing lipid rafts to form and recognition of several external structures, thus providing the necessary PRR oligomerization to induce a response. EPEC intimate attachment has indeed been demonstrated through lipid rafts, activating NF-κB and MAPK and production of IL-8 [51]. Co-evolved symbiont bacteria are limited from epithelial contact by IgA coating, antimicrobial peptide secretion and the mucus layer. These bacteria only become a threat following epithelial damage or reduced barrier defence, when they gain access to the epithelium and induce an immune response [42], [52]. Whilst our findings are based on study of the prototypical exemplars of EPEC [53] and EAEC [54], further study is needed to address any possible inter-strain variation and applicability to all EPEC and EAEC serotypes. Although the importance of epithelial contact has been replicated independently with an alternative EAEC serotype O104:H4 during a recent epidemic [55], implicating a generalised phenomena worth consideration in future vaccine design.

As well as host-microbial contact being important, another determinant of the response is time. We consistently found IL-8 to be induced earlier than CCL20, pointing to different signalling events. CCL20 induces recruitment of dendritic cell (DC) precursors [56] which in turn can prime either a tolerogenic or an inflammatory immune response by modulating T cell lineage differentiation [57]. Our data suggest that in early infection IL-8 is induced and initial neutrophil recruitment ensues. With persistent bacterial stimulus, CCL20 is also released to recruit DCs and initiate an adaptive immune response. The only strain to induce significant CCL20 at 4 h was the LifA mutant, identifying lymphostatin as a suppressor of early CCL20 production.

In conclusion, there are many layers of complexity to the interaction between bacteria and IECs. The ultimate response represents a balance between host activation and microbial suppression. The host ‘sees’ a bacterium by a combination of PRRs, with host-bacterial contact a key determinant of the IEC response. From the microbial perspective the external structures are essential for colonisation. The ability to make contact with the epithelium is also important, enabling the bacteria to down-regulate host responses, for example *via* the TTSS. The final outcome depends on bacterial surface structures and soluble secreted factors, PRR compartmentalisation and the location and duration of these interactions. Understanding the complexities of the host-microbial balance will contribute to improved vaccine design for infectious gastroenteritis; potentially swaying the balance towards ‘protective’ immunity.

## Materials and Methods

### Bacterial culture and induction of virulence factors

All bacterial strains (See Table S1) were first cultured on Brain Heart Infusion (BHI) agar plates at 37°C over 24 h. A single colony from this plate was then cultured overnight in 3 ml of BHI broth at 37°C without shaking. To induce expression of virulence genes, overnight cultures were subsequently diluted 1∶30 in DMEM; incubated at 37°C and 100 rpm, until an optical density (OD) at A_600_ of 0.6 was obtained. Growth in DMEM increases the production of LEE encoded virulence factors [58]. For all strains the corresponding colony forming units (CFU) for an OD of 0.6 was quantified *via* serial dilutions plated on BHI agar plates.

### Assessment of bacterial surface structures by negative staining Transmission Electron Microscopy (TEM)

Negative staining TEM was used to visualise bacterial surface structures. Formvar/carbon coated copper/rhodium 100 mesh grids were coated with an activated bacterial culture for 1 min. The grid was air dried for 1 min and then coated with 1% aqueous ammonium molybdate (Agar Scientific, Stanstead, UK) for 10 s. Grids were air dried and examined using a Philips Transmission Electron Microscope (Philips CM120, Eindhoven, Netherlands) at an accelerating voltage of 80 kV. Each bacterial culture was examined in duplicate on three separate occasions. The size of the surface structures were estimated by direct measurement from printed micrographs using a measuring magnifier (×7) fitted with a 20 mm graticule (Polaron, Watford, Hertfordshire UK). Fimbriae and flagella were assessed visually based on previous morphological reports. Fimbriae were observed as hollow rod-like structures and were distinct from flagella. Type I and II fimbriae are 7–8 nm wide; and type III is 4–5 nm wide. Type I, II and III fimbriae can be approximately 0.5–2 µm long and are peritrichous. Type IV fimbriae are 10–20 µm long and are polar. Long polar fimbriae (LPF) are 2–10 µm long [59]. Flagella are not rigid rod structures and are wider (20 nm) and longer (>20 µm) than fimbriae as well as being monotrichous, lophotrichous or peritrichous [15]. Outer membrane vesicles (OMVs) are spherical vesicles composed of a bi-layer membrane with electron-dense luminal contents [14]. Exopolysaccharide (EPS) is a polymer structure with long polysaccharide chains and additional lipid or peptide groups [60].

### Cytokine stimulation

Recombinant cytokine interleukin-1β (IL-1β) was reconstituted according to the manufacturer's instructions (Sigma-Aldrich, Poole, UK). A concentration of 10 ng/ml of IL-1β was routinely used.

### Mannose inhibition of type 1 pili

For adhesion tests, 0.5% D-(+)-Mannose (Sigma-Aldrich, Poole, UK) was added to competitively inhibit any adhesion due to mannose-sensitive type 1 pili [23].

### Mammalian Caco-2 and HEp-2 cell culture

The Caco-2 cell line (ATCC: HTB-37) is widely used as a model system for the study of enterocytic function and as a model of the intestinal barrier [61]. The HEp-2 cell line (ATCC: CCL-23) is widely used as a model system to study the adhesion properties of human bacterial enteropathogens [62]. ∼1×10^6^ cells (Caco-2 or HEp-2) were seeded into a 6-well plate maintained at 37°C in a 5% CO_2_ in complete culture medium [DMEM supplemented with 10% heat inactivated foetal calf serum (FCS), 2 mM L–glutamine and 1% non-essential amino acids (Sigma, Poole, UK)]. Cells were grown until confluency was attained (Caco-2 for 7 days and HEp-2 cells for 48 h).

### Mammalian and bacterial co-culture

Cells were serum-starved overnight prior to stimulation. Cells were inoculated with bacteria grown to logarithmic phase (MOI ≈60) for 4 or 16 h at 37°C with 5% CO_2_. 10 ng/ml of IL-1β served as a positive control. The inoculation was carried out in complete culture medium. To prevent bacterial overgrowth 100 µg/ml gentamicin (Sigma-Aldrich, Poole, UK) was added 3 h post-infection [63].

Transwell® inserts (24 mm diameter, 0.4 µm pore size) were coated overnight with Rat tail collagen 10 µg/cm^2^ (Sigma-Aldrich, Poole, UK). Caco-2 cells were then seeded at a density of ∼1×10^6^ onto the inserts. Fresh medium was replenished every 2 days for both chambers. The transepithelial electrical resistance (TEER) was monitored with an epithelial tissue Voltohmeter resistance reader (World Precision Instruments, Stevenage, UK). Cells were allowed to differentiate for 14 days, when TEER values of 500–1000 Ωcm^2^ indicative of intact TJ formation were routinely achieved. The wells were then inoculated either apically or basolateraly.

### Fluorescent-actin staining (FAS) test

The FAS test allows visualisation of actin recruitment that occurs beneath bacterial attachment upon intimate adherence of EPEC [32]. Briefly following co-culture, cell monolayers (grown on13 mm glass cover slips) were washed three times with PBS to remove non-adherent bacteria and were then fixed with 4% formalin/PBS pH 7.4 for 20 min at RT°. The cell monolayers were permeabilised with 0.1% Triton-X 100/PBS for 30 min. The cells were incubated with 5 µg/ml FITC-conjugated phalloidin (Sigma-Aldridge, Poole, Dorset, UK) for 60 min at RT° followed by washing in PBS three times for 1 min and mounted onto glass slides using citifluor (Agar Scientific, Stanstead, UK). The cells were examined using a Zeiss UV microscope with 40× lens (numerical aperture 1.5) and images were acquired with a Zeiss Axiocam digital camera system (8-bit, 1300×1300 pixel-standard resolution) (Software-Image Associates, UK).

The number of localised or intimate adherent bacteria was quantified. Using inverse thresholding, grayscale images of both phase contrast and fluorescent micrographs were separately binarized. The adherent and intimate adherent bacteria appeared as dots that were quantified using the “analyze particle” tool of ImageJ (National Institutes of Health [NIH], Bethesda, USA). A ratio of adherent bacteria per cell was calculated. The total number of localised/intimate adherent bacteria per micrograph was divided by the total number of HEp-2 cells per micrograph. A total of three fields of view were selected at random for each condition per experiment and three independent experiments were conducted. The micrographs were thresholded under the same conditions.

### Cytokine specific gene expression

Following infection, cell monolayers were subjected to RNA extraction utilising TRIZOL (Invitogen, Paisley, UK) according to manufacturer's instructions. 5 µg of total RNA was reversed transcribed using the BioScript cDNA synthesis kit (Bioline, London, UK) according to the manufacturer's instructions. This was followed by RT-PCR, using previously characterised primers for human IL-8 and GAPDH (see Table S2) [64], [65]. Bands were visualized by ethidium bromide staining (Amresco, NBS Biologicals, Cambs, UK). Densitometric analysis of bands was conducted using the “gel analyzer” tool of ImageJ (NIH) software. Variations in mRNA levels were normalized to GAPDH expression and are expressed as fold induction compared to the uninfected control cells.

### Cytokine specific protein secretion

For the detection of IL-8 or CCL20 secretion into culture supernatants, IL-8 or CCL20 sandwich ELISA kits were used following the manufacturer's instructions (Quantikine, R&D, Abingdon, UK).

### Statistics

Statistical analyses were performed using SPSS 14 for Windows. Differences in gene or protein expression between control cells and stimulus (denoted as * on reaching statistical significance) and between wild type (WT) strains and their respective mutant strains (denoted as + on reaching statistical significance) were evaluated using a two-tailed Mann-Whitney *U*-test.

## Supporting Information

Figure S1 **EPEC BFP, TTSS and lymphostatin implicated in the balance between host induction and EPEC inhibition of IL-8 mRNA.** IL-8 and GAPDH gene expression of HEp-2 cells 4 h post-infection was determined by RT-PCR. Shown is a representative gel. Lane numbers: 1-unstimulated control, 2-IL-1β, 3-E69, 4-E69 + mannose, 5-E69 Smooth LPS, 6-E69 Rough LPS, 7-E69 ΔfliC, 8-E69 fliC^+^, 9-E69 31-6-1(1), 10-E69 JPN15, 11-E69 ΔecsN, 12-E69 ΔespA, 13-E69 ΔespF, 14-E69 ΔespC, 15-E69 ΔlifA, 16-mannose (17-reverse-transcriptase omitted).
(TIF)

Figure S2 **Phase-contrast and fluorescence micrographs showing control HEp-2 cells (a) and HEp-2 cells co-cultured with E69 WT (b) at 16 h.** Utilizing the fluorescent-actin staining test, each co-culture was examined in triplicate, shown is a representative of those seen. Complementarities of bacterial location [white arrow] and actin fluorescence [yellow arrow] was confirmed by simultaneously recording phase-contrast and fluorescence images. The images were analysed and the number of adherent bacteria quantified (see Figure 9).
(TIF)

Table S1 **Enteropathogenic E. coli [EPEC] species.**
(DOC)

Table S2 **Primers utilized in this study.**
(DOC)
