# Supplementary figures and images for: Bacterial-Epithelial Contact Is a Key Determinant of Host Innate Immune Responses to Enteropathogenic and Enteroaggregative Escherichia coli
Source: PLoS One. 2011 Oct 28;6(10):e27030. doi: 10.1371/journal.pone.0027030 (PMC3203933; doi:10.1371/journal.pone.0027030)

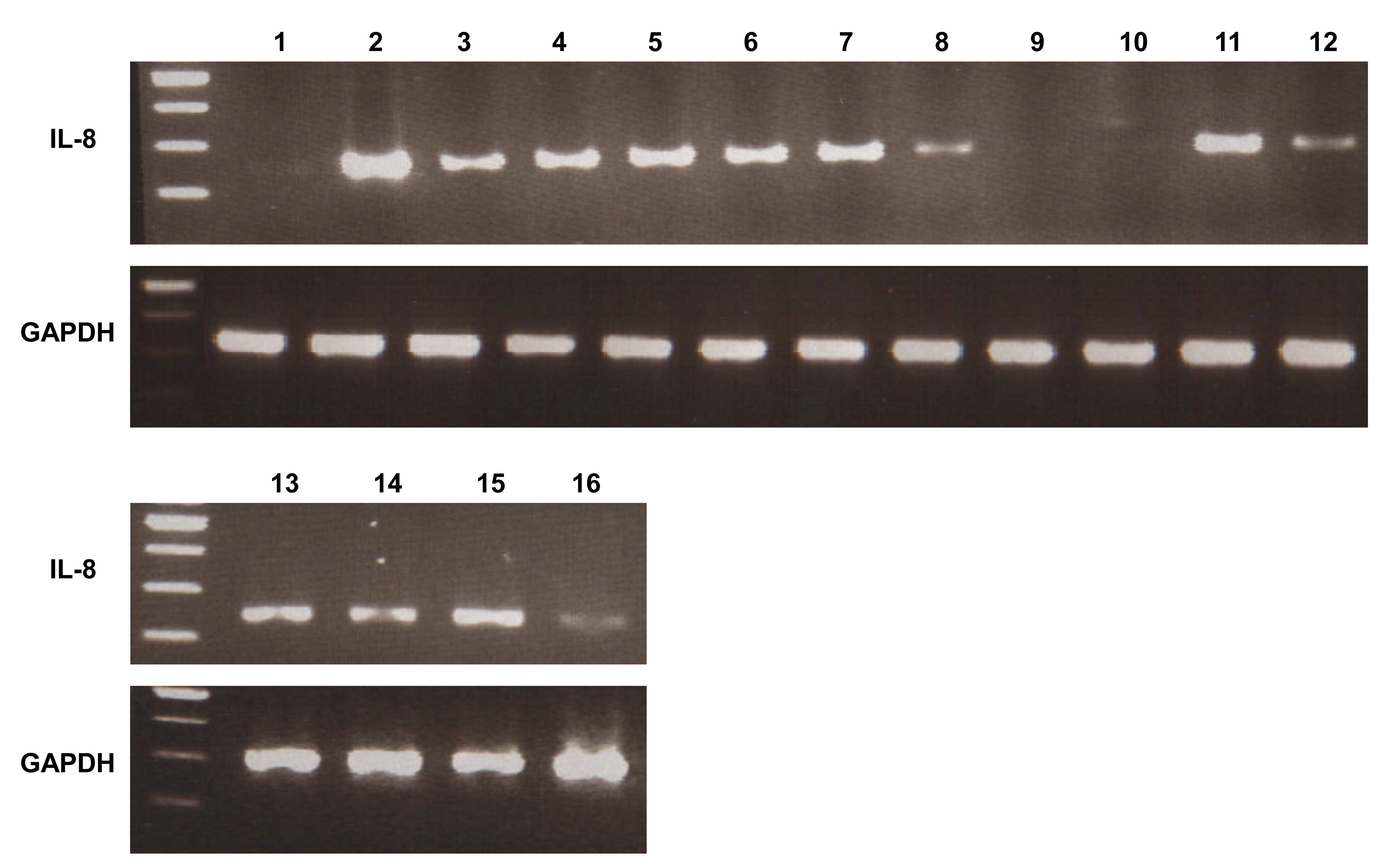

Supplement: Figure S1 — EPEC BFP, TTSS and lymphostatin implicated in the balance between host induction and EPEC inhibition of IL-8 mRNA. IL-8 and GAPDH gene expression of HEp-2 cells 4 h post-infection was determined by RT-PCR. Shown is a representative gel. Lane numbers: 1-unstimulated control, 2-IL-1β, 3-E69, 4-E69 + mannose, 5-E69 Smooth LPS, 6-E69 Rough LPS, 7-E69 ΔfliC, 8-E69 fliC+, 9-E69 31-6-1(1), 10-E69 JPN15, 11-E69 ΔecsN, 12-E69 ΔespA, 13-E69 ΔespF, 14-E69 ΔespC, 15-E69 ΔlifA, 16-mannose (17-reverse-transcriptase omitted). (TIF) [file pone.0027030.s001.tif]

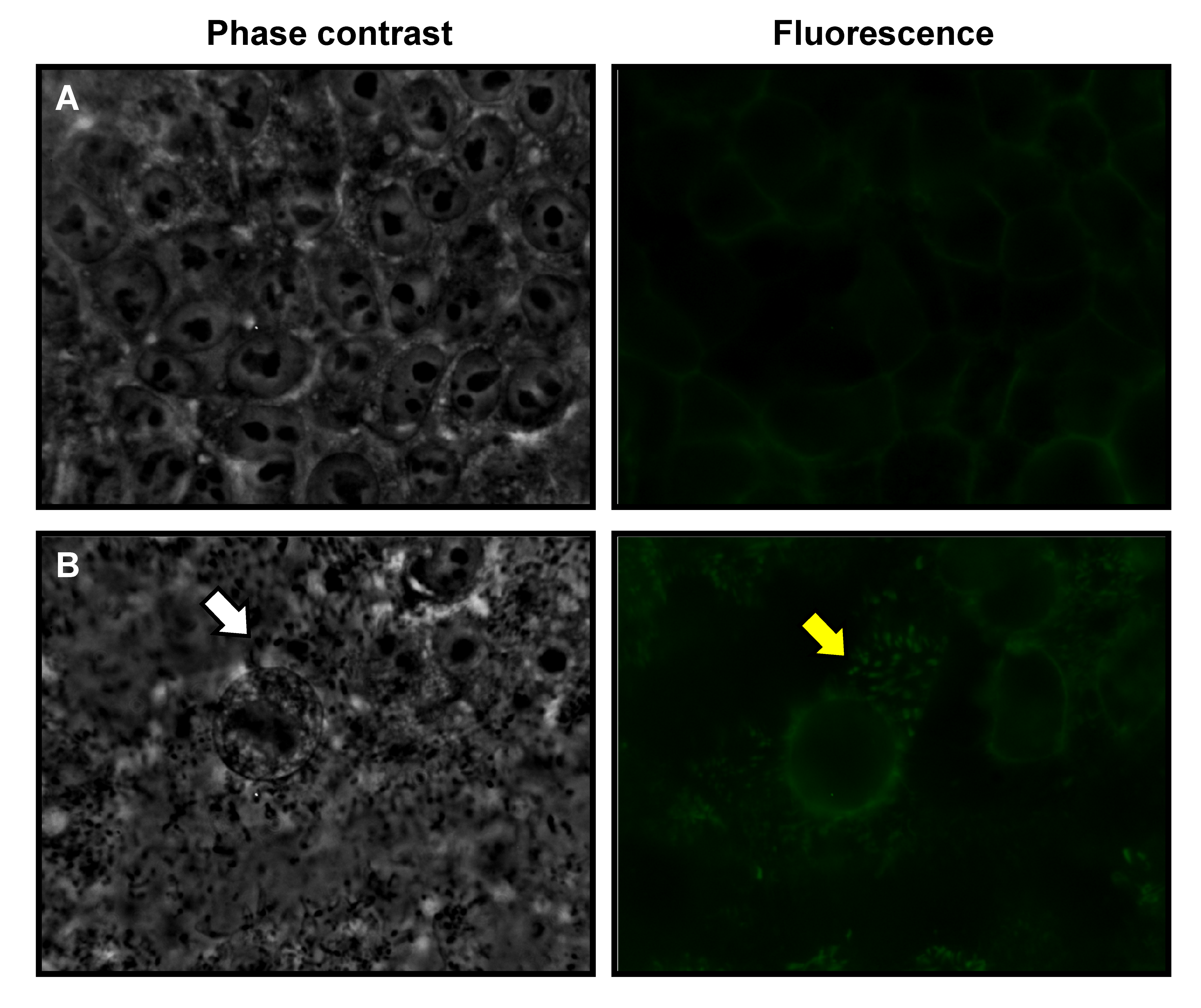

Supplement: Figure S2 — Phase-contrast and fluorescence micrographs showing control HEp-2 cells (a) and HEp-2 cells co-cultured with E69 WT (b) at 16 h. Utilizing the fluorescent-actin staining test, each co-culture was examined in triplicate, shown is a representative of those seen. Complementarities of bacterial location [white arrow] and actin fluorescence [yellow arrow] was confirmed by simultaneously recording phase-contrast and fluorescence images. The images were analysed and the number of adherent bacteria quantified (see Figure 9). (TIF) [file pone.0027030.s002.tif]
