# Supplementary material for: Bacterial-Epithelial Contact Is a Key Determinant of Host Innate Immune Responses to Enteropathogenic and Enteroaggregative Escherichia coli
Source: PLoS One. 2011 Oct 28;6(10):e27030. doi: 10.1371/journal.pone.0027030 (PMC3203933; doi:10.1371/journal.pone.0027030)
Supplement: Table S1 — Enteropathogenic E. coli [EPEC] species. (DOC) [file pone.0027030.s003.doc]

**Enteropathogenic *E. coli*** [EPEC] species

| **Strain/Serotype** | **Strain Characteristics** | **Source** | **Reference** |
| --- | --- | --- | --- |
| E2348/69 (E69 Wild Type)  Serotype: 0127:H6 | Fimbriae, Flagella, TTSS, effector proteins, Intimin | Prof James P Nataro | Levine MM et al. 1978 |
| Smooth LPS Serotype: 0127:H6 | Fimbriae, Flagella, TTSS, effector proteins, Intimin | Prof Ilan Rosenshine | Unpublished |
| Rough LPS Serotype: 0127:H6 | Fimbriae, Flagella, TTSS, effector proteins, Intimin  **Lacking** **o-polysacharide chains** | Prof Ilan Rosenshine | Unpublished |
| AGT01  E2348/69 *∆fliC*  Serotype: 0127:H6 | Fimbriae, TTSS, effector proteins, Intimin  **Flagella negative** | Prof James B Kaper | Girón JA et al. 2002 |
| AGT02  E2348/69 *fliC*^+^  Serotype: 0127:H6 | Fimbriae, Flagella, TTSS, effector proteins, Intimin  **Flagella enhanced** | Prof James B Kaper | Girón JA et al. 2002 |
| 31-6-1(1) (*TnphoA* in *bfpA*)  Serotype: 0127:H6 | Flagella, TTSS, effector proteins, Intimin  **No type IV fimbriae** | Prof Michael S Donnenberg | Donnenberg MS et al. 1992 |
| JPN15 (Cured of pMAR2)  Serotype: 0127:H6 | Flagella, TTSS, effector proteins, Intimin  **No type IV fimbriae**  **EAF- Bfp-** | Prof James B Kaper | Jerse AE et al. 1990 |
| E2348/69 + mannose  Serotype: 0127:H6 | Flagella, TTSS, effector proteins, Intimin  **No type I fimbriae** | Prof James P Nataro +  Sigma-Aldridge | Cravioto A et al. 1988 |
| CVD452  E2348/69 *∆escN*  Serotype: 0127:H6 | Fimbriae, Flagella, Intimin  **No TTSS so No secretion of effectors** | Prof James B Kaper | Jarvis KG et al. 1995 |
| UMD872  E2348/69 *∆espA*  Serotype: 0127:H6 | Fimbriae, Flagella, effector proteins, Intimin  **Diffuse secretion of effectors but no translocation** | Prof Michael S Donnenberg | Kenny B et al. 1996 |
| MAS111  E2348/69*∆espC*  Serotype: 0127:H6 | Fimbriae, Flagella, TTSS, Intimin  **No effector** | Prof Brett B Finlay | Stein M et al. 1996 |
| UMD874  E2348/69 *∆espF*  Serotype: 0127:H6 | Fimbriae, Flagella, TTSS, Intimin  **Effector Increased permeability** | Prof Michael S Donnenberg | Crane JK et al. 2001 |
| UMD704 E2348/69 *∆lifA/efaI*  Serotype: 0127:H6 | Fimbriae, Flagella, TTSS, Intimin  **Loss of ability to inhibit IL-2 expression** | Prof Michael S Donnenberg | Klapproth JMA et al. 2000 |

**Table S1: Bacterial strains utilized in this study**

**Enteroaggregative *Escherichia coli*** [EAEC] species

| **Strain/Serotype** | **Strain Characteristics** | **Test** | **Source** | **Reference** |
| --- | --- | --- | --- | --- |
| O42 Wild Type  Serotype: 044:H18 | Plasmid encoded cytotoxin, flagella, fimbriae | SEM ,  -VE TEM | Prof James P Nataro | Nataro JP et al. 1995 |
| O42 *∆fliC* (*pJP5603* in *fliC*)  Serotype: 044:H18 | Plasmid encoded cytotoxin, fimbriae  **Flagella negative** | -VE TEM | Prof James P Nataro | Steiner TS et al. 2000 |
| O42 *aafA* 3.4.14 (*TnphoA* in *aafA*)  O42 AAF/II  Serotype: 044:H18 | Plasmid encoded cytotoxin, flagella  **No AAF/II fimbria** | -VE TEM | Prof James P Nataro | Czeczulin et al. 1997 |
| O42 *aafB* (*pJP5603* in *aafB*)  Serotype: 044:H18 | Plasmid encoded cytotoxin, flagella  **AafB adhesin -ve** | -VE TEM | Prof James P Nataro | Elias et al. 1999 |

**Table S1 cont: Bacterial strains utilized in this study**
