# Supplementary material for: Bacterial-Epithelial Contact Is a Key Determinant of Host Innate Immune Responses to Enteropathogenic and Enteroaggregative Escherichia coli
Source: PLoS One. 2011 Oct 28;6(10):e27030. doi: 10.1371/journal.pone.0027030 (PMC3203933; doi:10.1371/journal.pone.0027030)
Supplement: Table S2 — Primers utilized in this study. (DOC) [file pone.0027030.s004.doc]

| Target gene | Sense primer | Anti-sense primer | Product size  In base pairs |
| --- | --- | --- | --- |
| IL-8 | ^41^ATG ACT TCC AAG CTG GCC GTG^62^ | ^308^TCT CAG CCC TCT TCA AAA ACT TCT C^332^ | 291 |
| GAPDH | ^630^CTA CTG GCG CTG GCA AGG CTG T^651^ | ^989^GCC ATG AGG TCC ACC ACC CTG CTG^966^ | 358 |

**Table S2: primers utilized in this study**
